# Supplementary material for: Insertion of Transposable Elements in AVR-Pib of Magnaporthe oryzae Leading to LOSS of the Avirulent Function
Source: Int J Mol Sci. 2023 Oct 24;24(21):15542. doi: 10.3390/ijms242115542 (PMC10650890; doi:10.3390/ijms242115542)
Supplement: Supplementary file 1 [file ijms-24-15542-s001.zip › Supplementary materials Figures S1-S7.pdf]

Supplementary materials

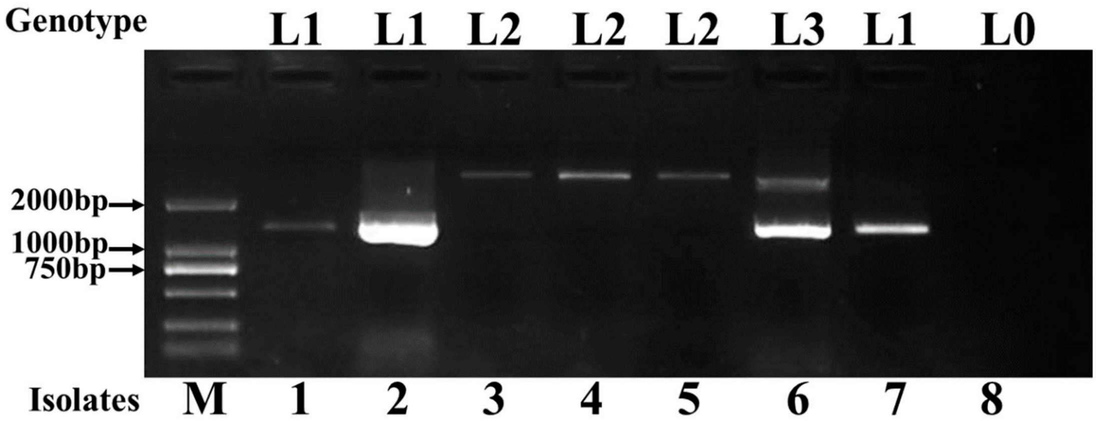

Figure S1. Detected alleles of *AVR-Pib* by PCR assays. The amplicons of *AVR-Pib*F1/R1 were used to distinguish four alleles: L1 and L2 (one fragment of different size), and L3 (two fragments of L1 and L2), L0 (no amplification); The isolates 1 to 8 are YJW-1-1d②, YJW-1-1e②, YJW-2-1a, YJW-2-1b, YJW-2-1c, YJW-2-1d, YJW-2-1e, and D-1-1g, respectively. M: DNA marker DL2000.

L1 indicates the *AVR-Pib* genotype with the expected size (1231bp), L2 and L3 indicates the *AVR-Pib* genotype with TE insertion (L2 with 3100bp, L3 with both of 1231bp and 3100bp).

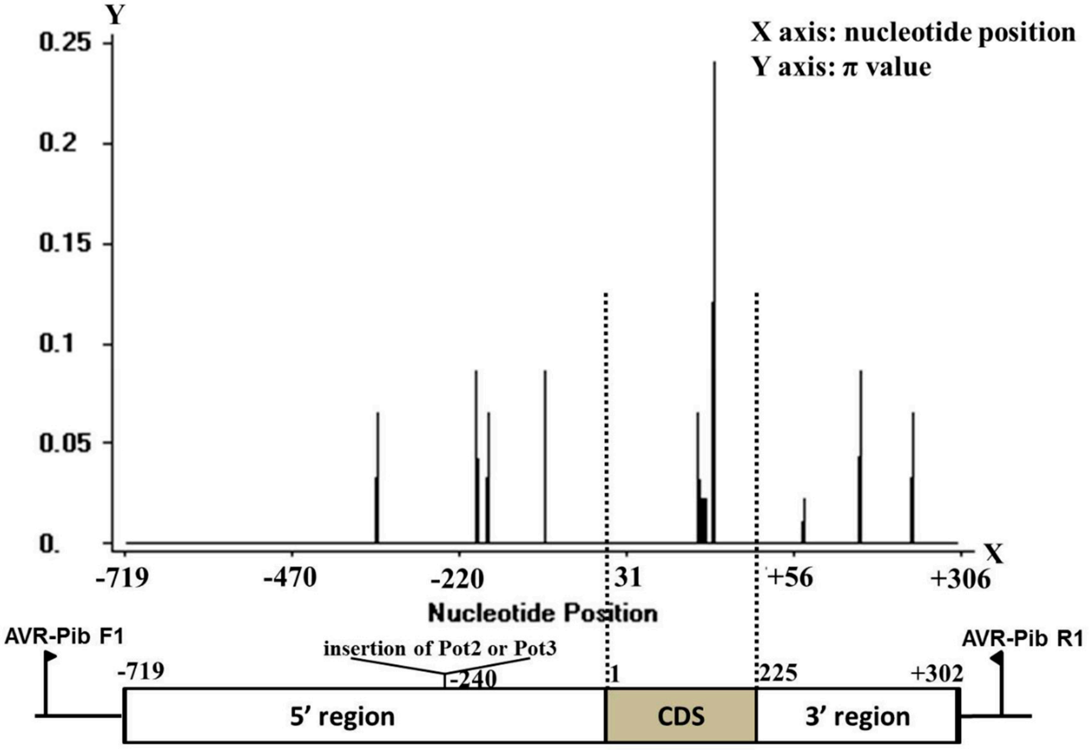

Figure S2. Diversification of *AVR-Pib* in avirulent isolates. Distribution of variation of the *AVR-Pib* alleles was analyzed using sliding window. X-axis shows the distribution of variation within the full region, including signal peptide and exon of *AVR-Pib*. Lower pane indicates the corresponding schematic presentation of the signal peptide and exon of *AVR-Pib*. Window length: 1; Step size: 1. π value corresponds with the level of variation at each site because it is the sum of pair-wise differences divided by the number of pairs within the population.

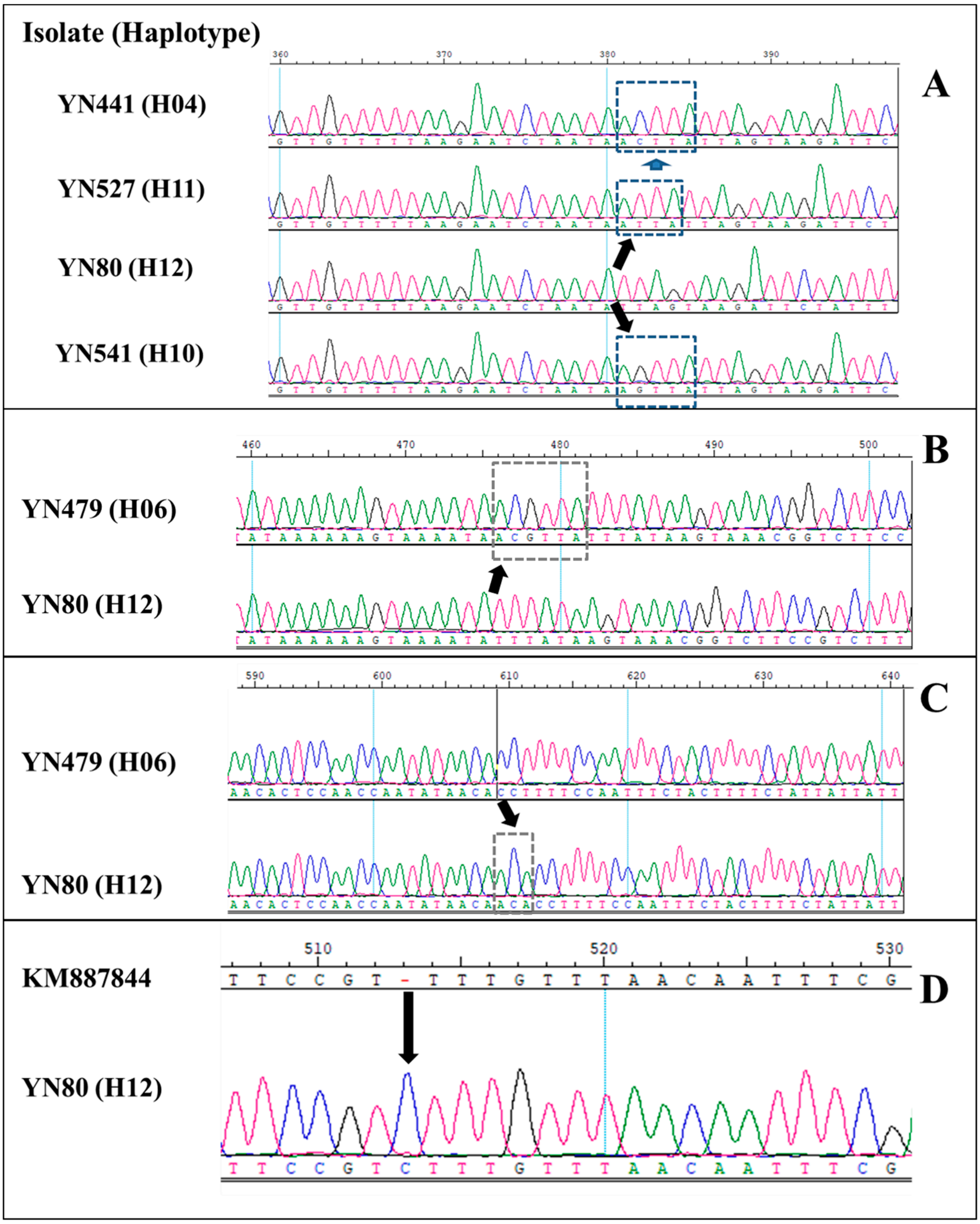

Figure S3. Part of insertion of sequences of *AVR-Pib*. **A,** indicates ACTTA, AGTTA, ATTA insert, respectively; **B,** indicates ACGTTA insert; **C,** indicates ACA insert; **D,** indicates C insert.

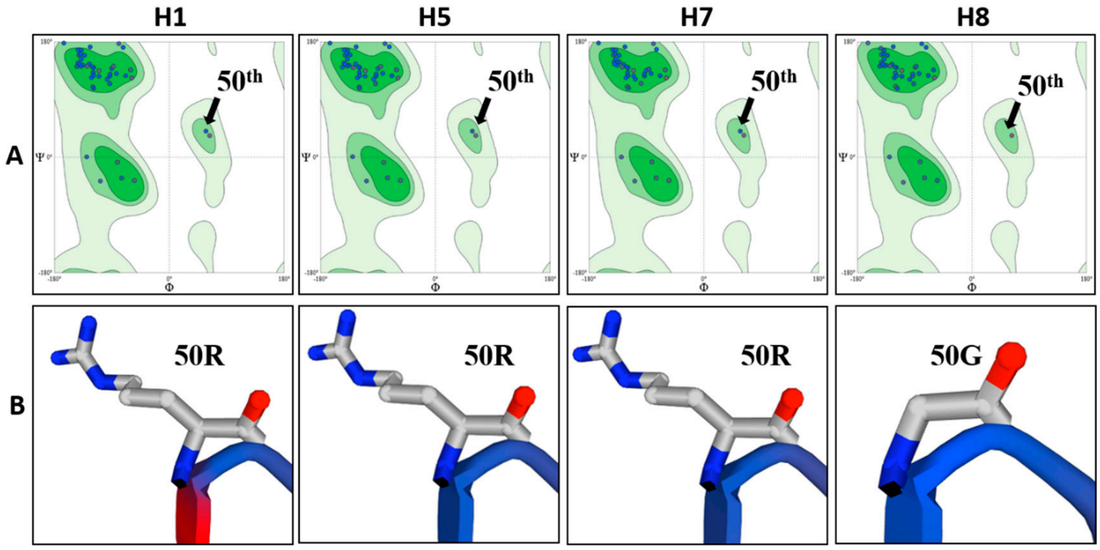

Figure S4. SWISS-MODEL homology modelling, built with PROMOD v. 3.70, method X-ray. Amino acid variations at R50G in H1 (samed with KM887844, H2, H3, H4, H9, H10, and H11), H5 (samed with H6), H7, and H8 protein haplotypes of AVR-Pib, respectively. **A,** Ramachandran Plots. **B,** the 50th amino acid of AVR-Pib.

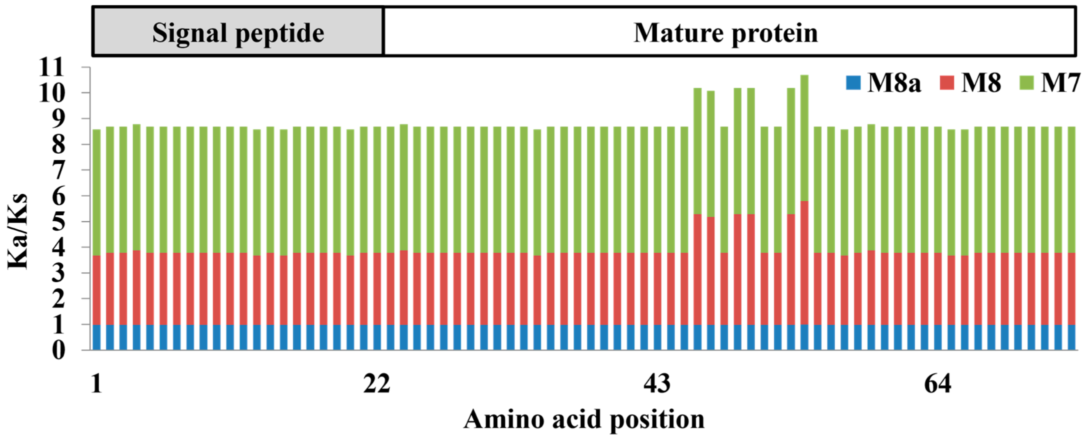

Figure S5. Sliding window of positive-selection sites of the AVR-Pib alleles under M8, M8a, and M7 models. The Y-axis indicates the ratio of the rate of nonsynonymous substitution (Ka) to the rate of synonymous substitution (Ks) (Ka/Ks); the X-axis indicates the position of the AVR-Pib amino acids in the site.

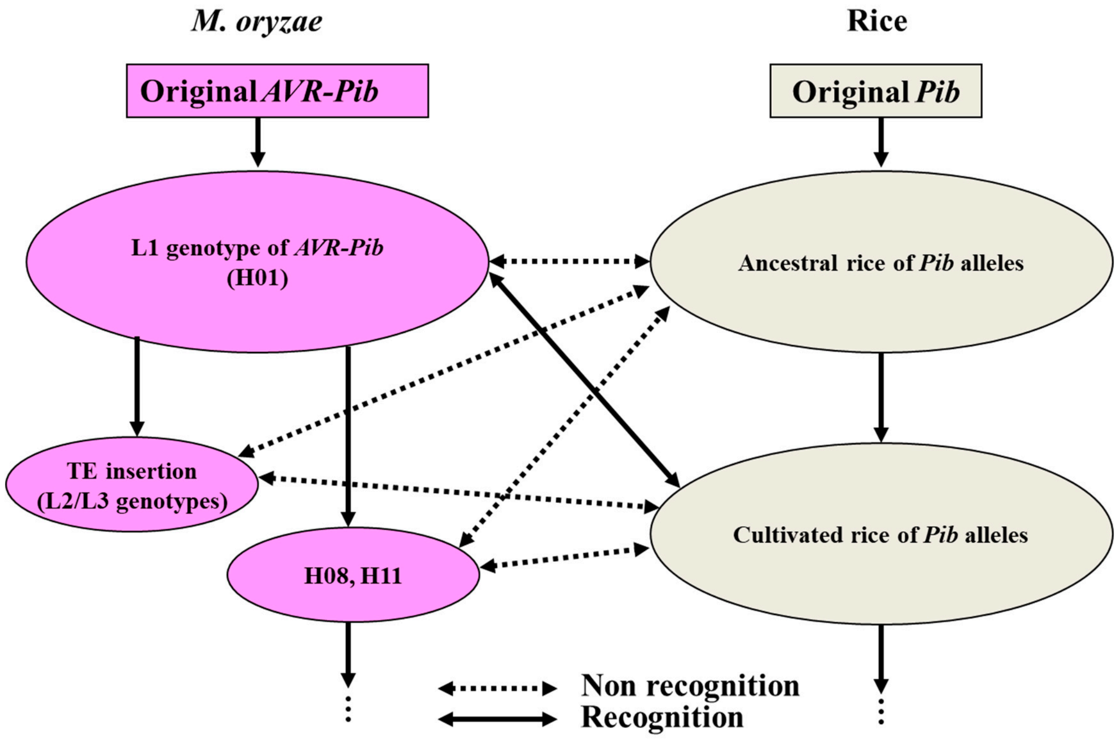

Figure S6. Possible scenario for *M. oryzae AVR-Pib* alleles-rice *Pib* alleles interactions and co-evolution. *AVR-Pib* homolog L1 genotypes (H01) were derived from an ancestral *M. oryzae* gene. L1 genotypes are cannot recognized by *Pib* in wild rice. In response to this situation, a 87bp insertion in exon1 of *Pib* in cultivated rice, evolved that can recognize L1 genotypes (H01) of *AVR-Pib* alleles. Then, another *AVR-Pib* alleles with TE insertion (L2/L3 genotypes), H08 and H11, were derived that cannot be recognized by *Pib* in cultivated rice.

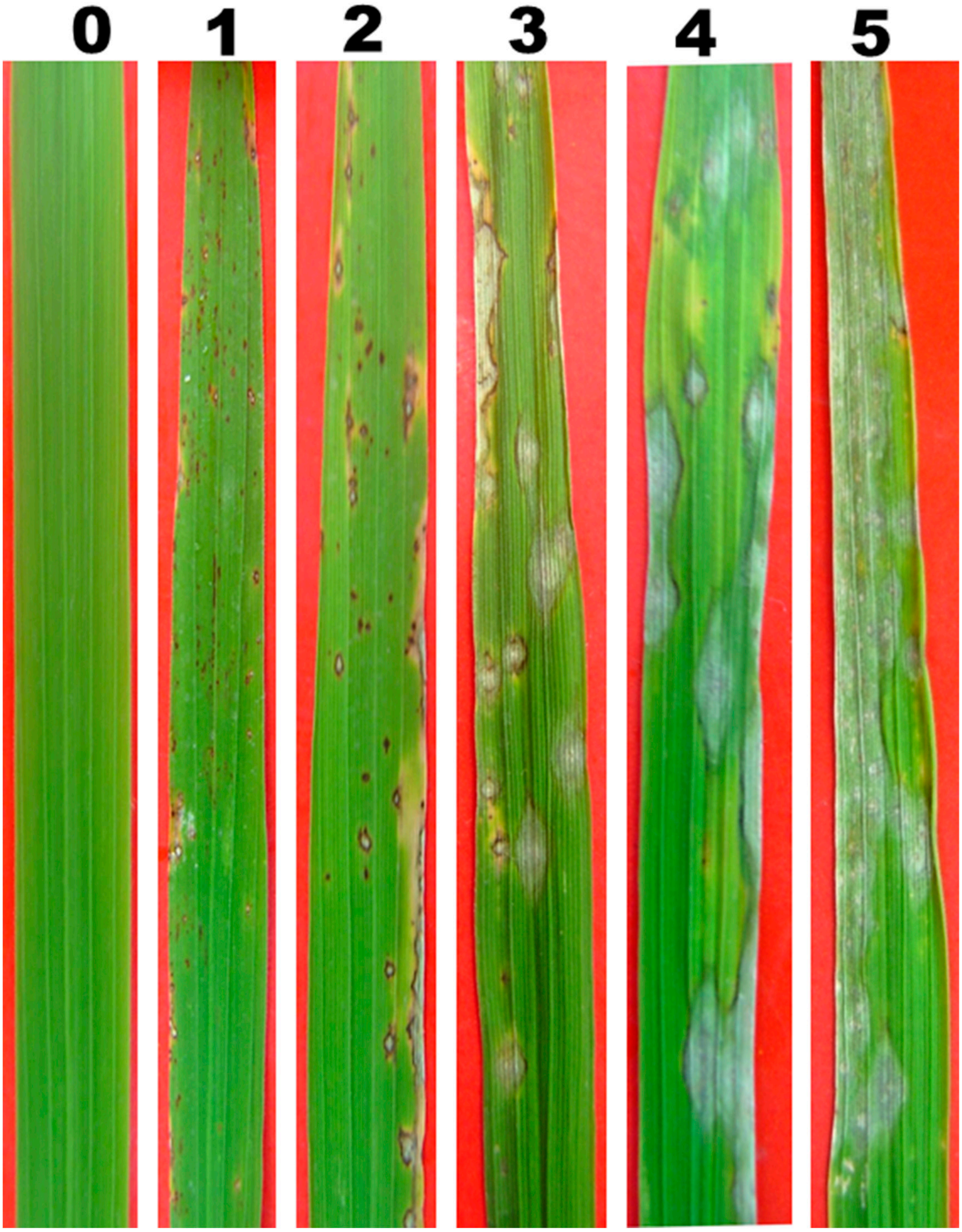

Figure S7. Disease reaction of *Magnapothe oryze* isolate on rice leaves. Number of 0 to 5 on the label on top of the Figure indicates 0-to-5 disease scale. A value of 0 to 1 is classified as “resistant”, 2 denotes “moderately resistant”, and 3 to 5 is classified as “susceptible”.
