# Supplementary material for: Insertion of Transposable Elements in AVR-Pib of Magnaporthe oryzae Leading to LOSS of the Avirulent Function
Source: Int J Mol Sci. 2023 Oct 24;24(21):15542. doi: 10.3390/ijms242115542 (PMC10650890; doi:10.3390/ijms242115542)
Supplement: Supplementary file 1 [file ijms-24-15542-s001.zip › Supplementary materials-Table S1-S3.pdf]

Supplementary materials

Table S1 Tajima's Neutrality Test of *AVR-Pib* in *M. oryzae*[a]

m	*S*	π	*D*
126	6	0.00164	-1.61687 (NS, 0.10>P>0.05)

[a] The analysis involved 126 nucleotide sequences of *AVR-Pib*. *m* indicates number of sequences, *S* indicates number of segregating sites, π indicates nucleotide diversity, and *D* is the Tajima test statistic. Tajima's *D*: -1.61687, Statistical significance: Not significant, 0.10>P>0.05.

Table S2 Genotype of *AVR-Pib* in isolates from different hosts

Species	Host	Total isolates	Genotype[a]			
			L0	L1	L2	L3
Wild rice	*Oryza rufipogon*	18	4	8	5	1
Banana	*Musa nana* Lour.	6	6	0	0	0
Wheat	*Triticum aestivum* Linn[b]	2	1	1	0	0
Weed	*Digitaria sanguinalis*	1	1	0	0	0
	Eleusine indica	1	0	1	0	0
	Eleusine coracana	1	0	1	0	0
	Lolium perenne Linn[b]	2	2	0	0	0
	Setaria viridis (Linn.) Beauv.[b]	1	1	0	0	0
	Total isolates	32	15	11	5	1

[a] L1 and L2 (one fragment of different size), and L3 (two fragments of L1 and L2), L0 (no amplification).

[b]The sequences come from Genbank.

Table S3 Lists accessions of *Pib* were obtained from GenBank used in this study.

Species	No.	Accession	Sub-species	Nucleotide sequences of exon1 of *Pib* used for phylogeny	Partial nucleotide sequences of exon3 of *Pib* used for phylogeny	Note
**Wild rice**	1	DQ317976.1	*O. longistaminata*	√		
	2	DQ317977.1	*O. meyeriana*	√		
	3	DQ298751.1	*O. officinalis*	√		
	4	DQ317975.1	*O. rufipogon*	√		
	5	DQ298750.1	*O. rufipogon*	√		
	6	DQ317978.1	*O. rufipogon*	√		
	7	EF642440.1	*O. rufipogon*		√	
	8	EF642443.1	*O. rufipogon*		√	
	9	EF642442.1	*O. rufipogon*		√	Pseudogene
	10	EF642441.1	*O. rufipogon*		√	Pseudogene
	11	EF642422.1	*O. nivara*		√	Pseudogene
Oryza sativa	12	KX791056.1	*Indica*	√		
	13	AB013449.2	*Japonica*	√		
	14	JN564623.1	*Indica*	√	√	
	15	JN564624.1	*Indica*	√	√	
	16	JN564625.1	*Indica*	√	√	
	17	AB013448.1	*Japonica*	√	√	
	18	AB026839.1	*O. sativa*	√	√	
	19	KR527239.1	*O. sativa*	√	√	
	20	KR527238.1	*O. sativa*	√	√	
	21	KR527240.1	*O. sativa*	√	√	
	22	KR527242.1	*O. sativa*	√	√	
	23	KR527241.1	*O. sativa*	√	√	
	24	KR527237.1	*O. sativa*	√	√	
	25	KR527234.1	*O. sativa*	√	√	
	26	KR527235.1	*O. sativa*	√	√	
	27	KR527233.1	*O. sativa*	√	√	
	28	KR527226.1	*O. sativa*	√	√	
	29	KR527227.1	*O. sativa*	√	√	
	30	KR527228.1	*O. sativa*	√	√	
	31	KR527229.1	*O. sativa*	√	√	
	32	KR527225.1	*O. sativa*	√	√	
	33	KR527224.1	*O. sativa*	√	√	
	34	KR527223.1	*O. sativa*	√	√	
	35	KR527222.1	*O. sativa*	√	√	
	36	KR527236.1	*O. sativa*	√	√	Pseudogene
	37	KR527231.1	*O. sativa*	√	√	Pseudogene
	38	KR527230.1	*O. sativa*	√	√	Pseudogene

39	KR527232.1	*O. sativa*	√	√	Pseudogene
40	EF642438.1	*Indica*		√	
41	EF642437.1	*Indica*		√	
42	EF642428.1	*Indica*		√	
43	EF642424.1	*Indica*		√	Pseudogene
44	EF642423.1	*Indica*		√	Pseudogene
45	EF642435.1	*Japonica*		√	
46	EF642426.1	*Japonica*		√	
47	EF642429.1	*Japonica*		√	
48	EF642427.1	*Japonica*		√	Pseudogene
49	EF642431.1	*Japonica*		√	Pseudogene
50	EF642434.1	*O. sativa*		√	
51	EF642436.1	*O. sativa*		√	
52	KR527245.1	*O. sativa*		√	Pseudogene
53	KR527244.1	*O. sativa*		√	Pseudogene
54	KR527243.1	*O. sativa*		√	Pseudogene
55	KR527246.1	*O. sativa*		√	Pseudogene
56	EF642433.1	*O. sativa*		√	Pseudogene
57	EF642432.1	*O. sativa*		√	Pseudogene
